# Intravoxel incoherent motion diffusion-weighted imaging in differentiating uterine fibroid from focal adenomyosis: initial results

**DOI:** 10.1186/s40064-015-1635-x

**Published:** 2016-01-04

**Authors:** Tao Tian, Guo-Fu Zhang, He Zhang, Hui Liu

**Affiliations:** 1 Department of Radiology, Shanghai No. 9 Hospital, School of Medicine, Shanghai Jiaotong University, Shanghai, People’s Republic of China; 2 Department of Radiology, Obstetrics and Gynecology Hospital, Fudan University, No. 419 Fang xie Road, Shanghai, 200011 People’s Republic of China; 3 Collaboration Scientist, MR Business Group, Healthcare Sector, Siemens Medical Solutions Ltd., Shanghai, People’s Republic of China

**Keywords:** Intravoxel incoherent motion, Diffusion-weighted imaging, Uterine fibroid, Focal adenomyosis

## Abstract

To evaluate the performance of intravoxel incoherent motion (IVIM)-diffusion-weighted imaging (DWI) in differentiating uterine fibroids from focal adenomyosises. Twenty-five uterine fibroids and 21 focal adenomyosises prospectively underwent IVIM-DWI examination prior to surgery. Four parameters including apparent diffusion coefficient total values (ADCtot), true diffusion coefficient (*D*), pseudodiffusion coefficient (D*) and perfusion fraction (*f*) derived from IVIM-DWI images were separately calculated and compared across four groups. There was a statistically significant difference in IVIM-derived *f* parameter between fibroid and focal adenomyosis (*p* = 0.01) and control group (*p* = 0.02). Uterine fibroids gave higher coefficient of variation (CV) of all IVIM-derived parameters than focal adenomyosises. IVIM-DWI could improve the sensitivity and specificity of detecting focal adenomyosis to 100 and 92.6 %, respectively. IVIM-*f* parameter could be potentially used to better distinguish uterine fibroid from focal adenomyosis. The higher CV of IVIM-derived parameters with acceptable range is often observed in the diseased group.

**Electronic supplementary material:**

The online version of this article (doi:10.1186/s40064-015-1635-x) contains supplementary material, which is available to authorized users.

## Background

Adenomyosis was firstly defined as benign invasion of endometrium in the myometrium, producing a diffusely enlarged uterus by Bird et al. in 1972(Bird and Manalo-Estrella 1972; Garcia and Isaacson 2011). It commonly affects premenopausal women and is associated with clinical manifestations similar to uterine fibroids (Azziz 1989), which are the most common tumor of the reproductive tract in women (Bulman et al. 2012). It is necessary to accurately differentiate uterine fibroid from focal adenomyosis owing to various therapeutic approaches.

With the advantages of the superb soft tissue resolution and no radiation, magnetic resonance imaging (MRI) has been widely performed to image pelvic diseases in clinical unit, especially for indeterminate masses on ultrasound (Sala et al. 2013). In recent studies, diffusion weighted imaging (DWI) has been used to distinguish malignant tumors from benign gynaecological diseases with promising results (Zhang et al. 2012; Thomassin-Naggara et al. 2013). It has been recognized that the calculated ADC value from lower *b* value images is more sensitive to capillary perfusion, representing motion of intravascular water protons within imaging voxels (Koh and Orton 2011; Takahara and Kwee 2012). Thus, IVIM approach proposed by Le Bihan et al. (1986), by using biexponential analysis, could integrate both tissue perfusion and diffusion effects in DWI images. Three parameters derived by IVIM, named as *D* (true diffusion coefficient), D* (pseudodiffusion coefficient) and *f* (perfusion fraction), are quantitative indexes used to reflect diffusion and perfusion changes in various tissues, i.e., head, liver, pancreas, colon, uterus and prostate (Lemke et al. 2009; Shinmoto et al. 2012; Sumi et al. 2012; Chiaradia et al. 2014; Doblas et al. 2013; Bisdas et al. 2013; Lee et al. 2014a).

To date, application of IVIM to image female pelvic diseases is still limited. The purpose of this study was to determine whether IVIM models could be explored to discriminate uterine fibroid from focal adenomyosis.

## Results and discussion

Finally, a total of 56 consecutive qualified subjects were recruited into the studied group, including 21 participants (25–52 years of age; average age, 37.9 ± 7.3) in focal adenomyosis group, 25 (28–69 years of age; average age, 44.4 ± 10.6) in uterine fibroid group and 10 with normal uterine structure as control group (24–69 years of age; average age, 40.9 ± 10.5). Others (23 endometrium cancer, 40 cervical cancer, 1 uterine sarcoma and 10 with unavailable patients’ consent,6 with claustrophobia and 7 with no final histological diagnosis) were excluded. The average SMR for both fibroids and focal adenomyosis was described in additional file 1: Figure S1. The details of SNR at varying b values DWI images were summarized in additional file 2: Figure S2.

**Fig. 1:**
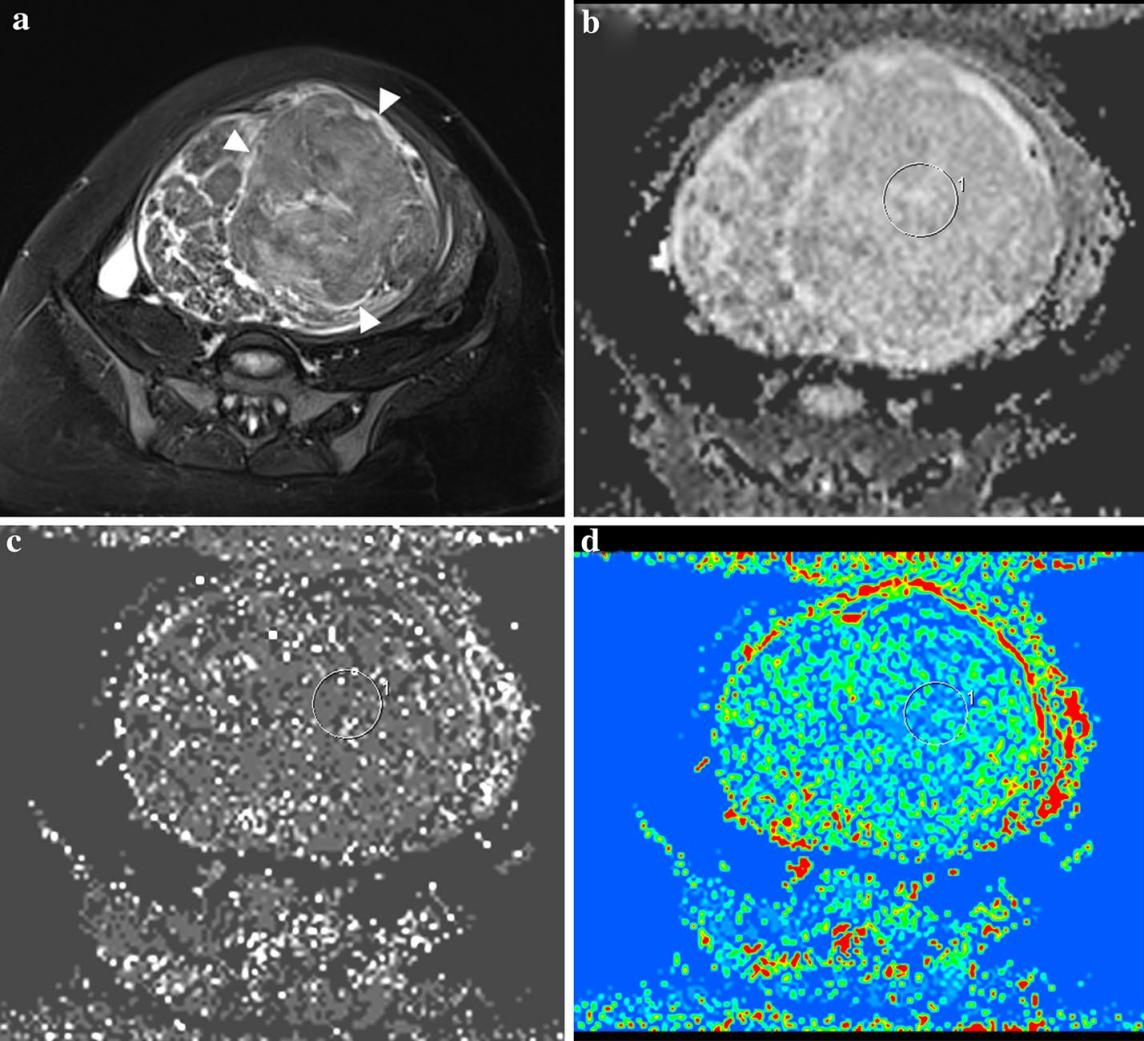
A 38-year-old patient with histologically proven uterine fibroid. **a** Axial FS T_2_WI reveals a giant mass occupying the main body of uterine (*arrowhead*); **b** *D* map shows reduced *D* value (2.138 ± 0.257 × 10^−3^ mm^2^/s); **c** D* map displays increased D* value (12.28 ± 14.3 × 10^−3^ mm^2^/s); **d** *f* map is 0.041 ± 0.041. (In this case, the signal decay curve generated by IVIM-DWI is shown in Fig. 4)

**Fig. 2:**
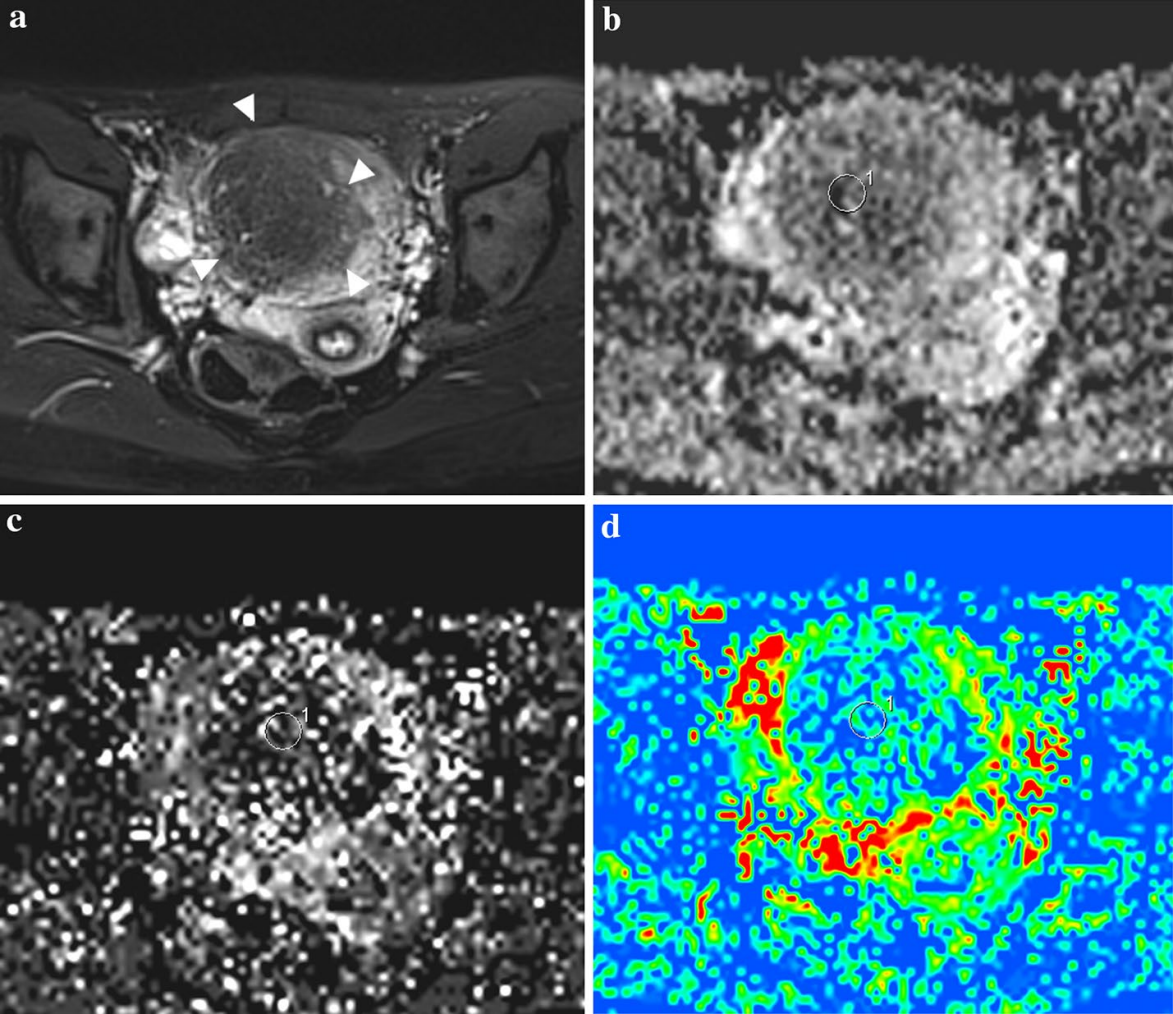
A 25-year-old patient with histologically proven focal adenomyosis. **a** Axial FS T_2_WI reveals a oval mass with main hypointensity signal occupying the myometrium and the junctional zone (*arrowhead*); **b** *D* map shows the mass is homogeneously isointensity signal with the *D* value of 0.515 ± 0.358 × 10^−3^ mm^2^/s; **c** D* map displays the D* value is 18.8 ± 19.9 × 10^−3^ mm^2^/s; **d** the *f* map is 0.114 ± 0.101. (In this case, the signal decay curve generated by IVIM-DWI is shown in Fig. 4)

The mean values of IVIM parameters for uterine fibroid (Fig. 1) were: ADCtot = 1.31 ± 0.43(×10^−3^ mm^2^/s), *D* = 1.12 ± 0. 43 (×10^−3^ mm^2^/s), D* = 15.9 ± 5.0 (×10^−3^ mm^2^/s), *f* (%) = 10.5 ± 6.3, respectively; for focal adenomyosis (Fig. 2) were: ADCtot = 1.09 ± 0.14 (×10^−3^ mm^2^/s), *D* = 0.95 ± 0. 13 (×10^−3^ mm^2^/s), D* = 16.8 ± 5.0 (×10^−3^ mm^2^/s), *f* (%) = 15.7 ± 3.6, respectively; for control group were: ADCtot = 1.24 ± 0.19 (×10^−3^ mm^2^/s), *D* = 1.18 ± 0. 21 (×10^−3^ mm^2^/s), D* = 18.6 ± 3.8 (×10^−3^ mm^2^/s), *f* (%) = 16.6 ± 8.0, respectively (Table 1).

**Table 1:** Comparison of IVIM parameters (mean ± standard deviation) between uterine fibroid, focal adenomyosis and control group

Group	N	ADCtot (10^−3^mm^2^/s)	*D* (10^−3^mm^2^/s)	D* (10^−3^mm^2^/s)	*f* (%)
Uterine fibroid	21	1.31 ± 0.4	1.12 ± 0.4	15.9 ± 5.5	10.5 ± 6.3
Focal adenomyosis	25	1.09 ± 0.1	0.95 ± 0.1	16.8 ± 5.0	15.7 ± 3.6
Control group	10	1.24 ± 0.2	1.18 ± 0.2	18.6 ± 3.8	16.6 ± 8.0

**Fig. 3:**
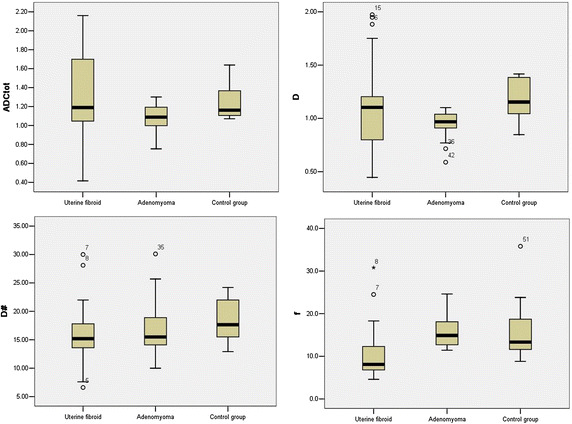
Box plots (*top* and *bottom of boxes* represent 25–75 ‰ of the data values; *line in box* represents median value; *circles* represents the outliers; *asterisk* represents extreme cases) of ADCtot (10^-3^ mm^2^/s) , *D * (10^-3^ mm^2^/s), D* (10^-3^ mm^2^/s) and *f* (%) in uterine fibroid, focal adenomyosis and control group. Note, *f* in uterine fibroid is significantly lower than focal adenomyosis (*p* = 0.01) and control group (*p* = 0.02)

**Fig. 4:**
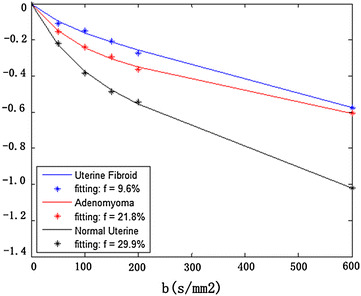
Biexponential fit of the signal decay in according with the varying *b* values in three selected samples. *Blue line* represents uterine fibroid (Fig. 1); *red line* represents focal adenomyosis (Fig. 2); *black line* represents the normal uterine. Note, IVIM-*f* in uterine fibroid significantly lows than that in both focal adenomyosis and control group

**Fig. 5:**
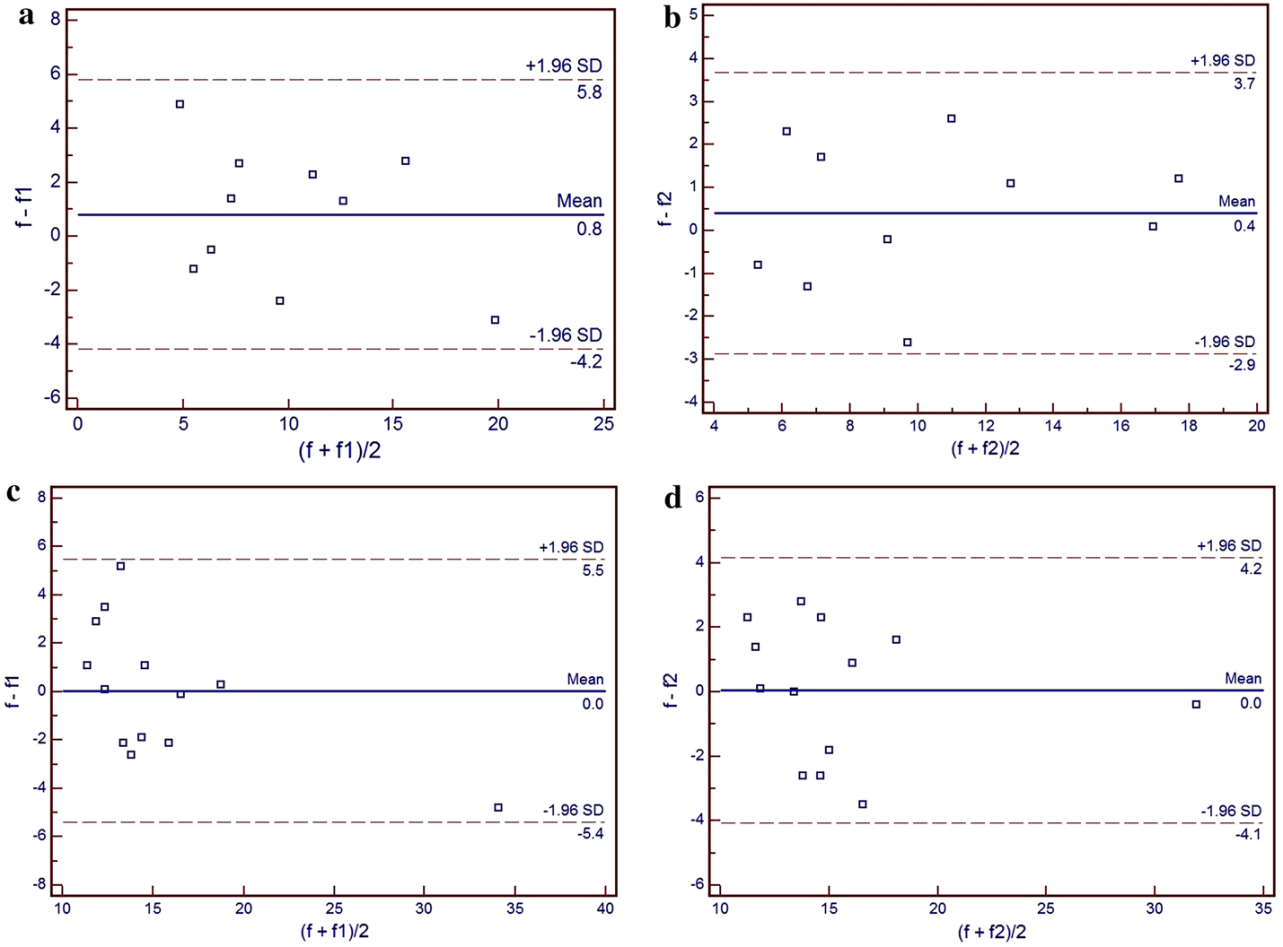
Bland–Altman plots estimate the interobserver reliability (**a**, **c**) and intraobserver repeatability (**c**, **d**) of the IVIM-*f* parameters in uterine fibroid (**a**, **b**) and focal adenomyosis (**c**, **d**). The differences in the *f* values between the first and the second measurements (y-axis) are plotted against the averages of them (x-axis), with mean difference and 95 % limits of agreement indicated

The statistically significant differences were only observed in *f* parameter between fibroid and focal adenomyosis (*p* = 0.01) and control group (*p* = 0.02) (Figs. 3, 4). The detailed significant differences of IVIM parameters at statistical level within three groups were listed in Table 2. Regarding the repeatability of the IVIM-based parameters, the CVs of ADCtot, *D*, D* and *f* between uterine fibroid and focal adenomyosis group were 0.31, 0.25, 0.17, 0.44 and 0.14, 0.19, 0.38,0.20, respectively. The CVs of the IVIM model parameters in uterine fibroid were relatively higher than focal adenomyosis group, while much higher than the control group (Table 3). The Bland–Altman plots demonstrated satisfactory results without any outliers outside the mean ± 1.96 SD boundaries in all cases, indicating a good agreement in both inter-observer reliability and intra-observer reproducibility (Fig. 5). On T_1_WI, both fibroid and focal adenomyosis appeared intermediate signals (similar with myometrium). On T_2_WI, most fibroids appeared as low signals (14/21), seven cases showed iso/hyper signals (similar with endometrium); for focal adenomyosis, all lesions in the studied samples were iso/hypo signals on T_1_WI and iso/hyper signals on T_2_WI. Overall, combining with IVIM-DWI information, the sensitivity and specificity of MRI for detecting focal adenomyosis were 100 and 92.6 %, respectively, which was higher than only with conventional MRI reading session (Table 4).

**Table 2:** The statistically significant difference (*p* value) of IVIM parameters within three groups

	ADCtot	*D* (10^−3^mm^2^/s)	D* (10^−3^mm^2^/s)	*f* (%)
Uterine fibroid and focal adenomyosis	0.072	0.146	0.836	0.010
Uteine fibroid and control group	0.829	0.890	0.347	0.020
Focal adenomyosis and control group	0.445	0.143	0.626	0.930

**Table 3:** Coefficient variations of IVIM parameters measurements in 11 subjects within three groups

Parameters	Uterine fibroid	Focal adenomyosis	Control group
ADCtot	0.31	0.14	0.004
*D* (10^−3^mm^2^/s)	0.25	0.19	0.004
D* (10^−3^mm^2^/s)	0.17	0.38	0.08
*f* (%)	0.44	0.20	0.16

**Table 4:** Diagnostic performance according to two kinds of MRI protocols

Protocol	SEN (%)	SPE (%)	PPV (%)	NPV (%)	ACC (%)
Conventional MRI	90.0 (18/20) (68.3–98.8)	88.5 (23/26) (69.9–97.6)	85.7 (18/21) (63.7–97.9)	92.0 (23/25) (74.0–99.0)	89.1 (41/46) (75.6–95.9)
Conventional MRI plus IVIM	100.0 (19/19) (82.4–100.0)	92.6 (25/27) (75.7–99.0)	90.5 (21/23) (69.6–98.8)	100.0 (25/25) (86.3–100.0)	95.7 (44/46) (85.5–98.8)

Numbers in parentheses are the data used to calculate the percentages. Numbers in brackets are 95 % confidence intervals; conventional MRI includes T1wi/T2wi/contrast-enhanced MRI

Both fibroid and adenomyosis are the most common benign condition of the uterus in women of reproductive age and often coexist with similar clinical complains (Jha et al. (2014). On MRI, these two etiologies could be easily differentiated based on imaging signs and specific lesion characters (Takeuchi and Matsuzaki 2011). Sometimes it is difficult to discriminate focal adenomyosis or small adenomoyoma from fibroids (Matsumoto et al. 2013). Considering various treatments for each etiology, accurate diagnosis is still needed prior to aggressive treatments. Here, we reported our preliminary experiences with IVIM-MRI approach in differentiation between uterine fibroid and focal adenomyosis in our institution. Our data showed that IVIM-*f* was a more robust index than IVIM-*D* and IVIM-D* parameter to discriminate uterine fibroid from focal adenomyosis with no overlap (*p* = 0.01).

DWI-MRI is a functional imaging technique that is now widely applied in categorizing suspected lesions, staging malignancies and monitoring therapeutic effects (Sala et al. 2013; Zhang et al. 2012; Stamatopoulos et al. 2012; Lee et al. 2014b; Zhang et al. 2014). By using multiple *b* values, IVIM-DWI could potentially incorporate both perfusion and diffusion information to describe the tissue signal attenuation with mathematical model fitting (Koh and Orton 2011; Takahara and Kwee 2012; Le Bihan et al. 1986). In contrast to perfusion parameters derived from other techniques (i.e., dynamic contrast enhanced imaging), owing to the advantages of free-contrast and shorter acquisition time, IVIM-DWI has gained increasing attractions in clinically relevant application (Lemke et al. 2009; Sumi et al. 2012; Chiaradia et al. 2014; Doblas et al. 2013). Several recent studies with focus on IVIM-derived parameters in various tissues characterization have been published (Liu et al. 2013; Lu et al. 2013; Sumi and Nakamura 2014).

There are mainly four mathematical models including the monoexponential model, the stretched exponential model, the kurtosis model and the biexponential model to quantify DWI signal decay (Jambor et al. 2014; Merisaari and Jambor 2014). Among them, a monoexponential fit model is the simplest mathematical model to define signal decay with more robust parameter than the other three models (Takahara and Kwee 2012). In one study, the authors declared that the parameters calculated with monoexponential, kurtosis, and stretched-exponential models had better reliability and repeatability of the fitted parameters than the biexponential model (Merisaari and Jambor 2014). Our study corroborated this point that the CV of IVIM-derived parameters (*f*, *D*, D*) from the biexponential model was relatively large and may be more sensitive to noise.

In one study, Yang et al. investigated the value of DWI at 3.0-Tesla MR unit in the differentiating uterine adenomyosis from uterine fibroids, suggesting uterine adenomyosis demonstrated significantly higher mean ADC values than uterine leiomyoma (Yang et al. 2011). In our study, there was no difference in ADCtot values derived from IVIM images between uterine fibroid and focal adenomyosis (*p* = 0.072). Of note, in the studied fibroid group, IVIM-derived parameters have much larger variation compared with the other two groups. Inhomogeneous signals on T_2_WI (7/21) were more often observed in fibroid group, indicating some likely degeneration which may influence the final calculation.

Our results demonstrated that IVIM-*f* parameter (reflecting tissue microcapillary perfusion) could be a potential indicator in differentiating fibroid from focal adenomyosis (10.5 vs. 15.7 %, *p* = 0.01). These data well correlated with the histological results that proliferative ectopic endometrial tissues in the myometrium contains plenty of capillary vessels, increasing blood flow volume in the whole lesion. Further, IVIM images could also aid radiologists to improve their diagnostic performance in discriminating fibroid from adenomyosis before invasive procedure.

Inter-examination reproducibility is an important estimation of the reliability of IVIM as a clinically useful discriminator. In this study, *D* is much more reliable (19–25 %) compared with D* (17–38 %) and *f* (20–44 %), consistent with published results from another study (Lai et al. 2013). In general, these variations were acceptable, especially regarding the much lower CV of IVIM-derived indexes in the control group.

There were several limitations of this study. Firstly, the IVIM processing software we used here is not commercially standardized till now; the purpose of these acquisition protocols mainly apply for scientific research. Secondly, we selected six *b* values to acquire IVIM-DWI data, which was different with other studies. Theoretically, the choice of much lower *b* values may more accurately reflect perfusion sensitive signal attenuation (Koh and Orton 2011; Takahara and Kwee 2012). However, free-breathing technique was used to accommodate multiple *b* values in this study, thus, patients movements are unavoidable when increase the acquisition time length; SNR variations which may also be accordingly elevated, resulting in inaccurate signal measurements at multiple *b* values images. The total acquisition time of 3.5 min in this study was acceptable for all patients and the signal decay fitting line can also be roughly modeled (Fig. 4). Thirdly, ROIs were manually drawn and individually calculated on a case-by-case basis, and lack of standardization may effect on the final results. Finally, high-field MR unit (3 Tesla) has been gradually introduced into the clinical market. It is also needed to determine whether or not there is any difference in application of IVIM in uterine lesions between 1.5T and 3T MR unit.

## Conclusions

In summary, IVIM-*f* can be used as a quantitiative parameter to better differentiate uterine fibroid from focal adenomyosis. The higher CVs of IVIM-derived parameters with acceptable range are more often observed in the disease group than the control group.

## Methods

### Study subjects

This study was approved by our institutional review board. Patients or qualifying family members provided their written informed consent before participation. From March 2013 to June 2013, 143 consecutive patients with clinically suspected pelvic disease prospectively underwent MRI and IVIM examination. Laparotomy or laparoscopic surgery was performed to confirm the etiology of uterine diseases. The time interval between MRI and surgery was less than 1 month. Inclusion criteria were: (1) newly suspected uterine diseases; (2) no previous treatment history. Exclusion criteria were: (1) contraindication for MRI examinations; (2) uncooperative patients or unavailable of patient’s consents.

### Image acquisition

MR imaging was performed using a 1.5-T MR system (Magnetom Avanto, Siemens, Erlangen, Germany) with a phased-array coil. The routine MRI protocols used for the assessment of pelvic masses included the axial turbo spin-echo (TSE) T_1_-weighted imaging (T_1_WI, repetition time/echo time (TR/TE) = 550/10 ms), sagittal TSE T_2_-weighted imaging (T_2_WI, TR/TE = 4000/83 ms) and axial/sagittal TSE fat-suppressed T_2_WI (FS T_2_WI, TR/TE = 8000/83 ms). Diffusion-weighted imaging (DWI) using an echo-planar imaging two-dimensional (EP2D) sequence in free-breath performed in the axial plane with parallel acquisition technique (GRAPPA acceleration factor of two) by using six *b* values (0, 50, 100, 150, 200 and 600 s/mm^2^). The details of acquisition parameters were as follows: TR = 4000 ms, TE = 78 ms, field of view (FOV) = 280 mm, slice thickness = 5 mm, bandwidth = 1726 Hz/Pixel, fat suppression with spectral pre saturation attenuated inversion recovery (SPAIR) technique. Average ADC map was automatically generated. The image resolution generated from IVIM yielded an approximate voxel size of 2.0 × 2.0 ×5.0 mm with a total examination time of three and a half minutes.

### DW-MRI data analysis

The IVIM model is described by the equation below, where *D* and D* are the diffusion parameters related with molecular diffusion and with the incoherent microcirculation respectively, S is the mean signal intensity and *f* is perfusion fraction, i.e., the fraction of the pseudo-diffusion (or perfusion) correlated with microcirculation:

$$\frac{S(b)}{{S_{0} }} = (1 - f)\exp ( - b*D) + f*\exp ( - b*(D + D^{*} ))$$

where *S*_i_ is the signal at *b* = b_i_, *S*_0_ is the baseline signal, where *b* = 0; *D* is the slow diffusion decay associated with extravascular water molecules’ motion; D* is the fast diffusion decay associated with the intravascular water molecules’ motion; and *f* is the fraction perfusion compartment in the two compartments.

A work in progress post-processing program is used to fit the above IVIM bi-exponential model to generate three parametric images (*D*, D* and *f*) using two segment method, where an initial estimation of *D* using a reduced set of *b*-values larger than a predetermined value (in our case, *b* = 200 is used.) and then using the resulting *D* as a fix parameter to fit the missing parameters similar to what was described in (Luciani et al. 2008). In addition, we estimated the ADC of the mono exponential signal decay model:

$$S_{i} = S_{0} \exp ( - b_{i} ADC)$$

where S_i_, b_i_, and S_0_ are as defined above. The total ADC value (ADCtot) was then measured by using the entire range of *b*-value images on IVIM-map.

### Image data analysis

Firstly, all MRI image raw data were reviewed by two readers (H.Z., T.T.) blind to the final pathological results; the final conclusion was made with consensus reading. Four IVIM-derived parameters (ADCtot, *D*, D* and *f*) were separately measured in two sessions (with 3-month interval) for evaluating the reproducibility of data interpretation. ADCs were measured manually on commercially available post-processing workstation (Leonardo, Siemens, Germany) by one reader (H.Z.). The signal-muscle ratio (SMR) for each lesion at both T_1_WI and T_2_WI sequence and the signal–noise ratio (SNR) of DWI images at varying *b* values were calculated by the same reader (H.Z.). Regions of interest (ROI) with average circle area from 180 to 220 mm^2^ was placed into the mostly solid part of each lesion in both fibroid and focal adenomyosis group. For multiple lesions in one subject, we chose the largest one as the targeted lesion for the further evaluation.

### Statistical analysis

Numerical variables were expressed as the mean ± SD. The factor analysis within a set of measured variables across each parameter was validated by Tukey’s test. The repeatability of the IVIM results was tested by the CV; A Bland–Altman analysis was employed to analyze the agreement between the two measurements. The receiver operating characteristic (ROC) curve was calculated for each IVIM parameter in differentiating fibroid from adenomyosis. The diagnostic performance of MRI based on two series of protocols (conventional MRI and conventional MRI plus IVIM) were calculated as accuracy (ACC), sensitivity (SEN), specificity (SPE), positive predictive values (PPV), and negative predictive values (NPV), expressing as percentages [95 % confidence interval (CI)]. A p value less than 0.05 was considered statistically significant. SPSS (version 13.0, SPSS Inc., Chicago, USA) and MedCalc (version9.2.1.0, MedCalc Sofware, Ostend, Belgium) were used to perform statistical.

### Additional files

**Additional file 1: Figure S1.** Illustrating case of signal–noise-ratio calculation at various b values DWI images. A 29-year-old female with focal adenomyoma in the posterior wall of uterine. One reviewer (H.Z.) placed the ROI_1_ with average area 2.54 cm^2^ in the center of lesion. Similarly, on the same series of pictures, ROI_2_ with an average area of 2.80 cm^2^ indicating the noise signals was also placed on the background. The SNR values in ten cases in each group were calculated. To minimum the operator bias, only one experienced operator did the whole procedure.

**Additional file 2: Figure S2.** SNR values at various *b* values in three groups. The final SNR values for each group were 28.2 ± 9.2 at b = 0, 32.6 ± 4.3 at b = 50, 29.9 ± 0.8 at b = 100, 27.5 ± 2.1 at b = 150, 25.9 ± 4.3 at b = 200, 17.0 ± 1.0 at b = 600 for myoma and 32.1 ± 2.0 at b = 0, 49.3 ± 2.0 at b = 50, 48.9 ± 5.9 at b = 100, 47.9 ± 1.9 at b = 150, 41.4 ± 2.9 at b = 200, 30.7 ± 3.3 at b = 600 for adenomyoma and 45.8 ± 2.2 at b = 0, 63.3 ± 2.5 at b = 50, 52.5 ± 4.4 at b = 100, 46.7 ± 5.2 at b = 150,45.8 ± 3.5 at b = 200, 27.6 ± 3.3 at b = 600 for control group, respectively.

## Abbreviations

IVIM: intravoxel incoherent motion
DWI: diffusion weighted imaging
ADC: apparent diffusion coefficient
CV: coefficient of variation
MRI: magnetic resonance imaging
