# Supplementary figures and images for: Intravoxel incoherent motion diffusion-weighted imaging in differentiating uterine fibroid from focal adenomyosis: initial results
Source: Springerplus. 2016 Jan 4;5(1):9. doi: 10.1186/s40064-015-1635-x (PMC4700030; doi:10.1186/s40064-015-1635-x)

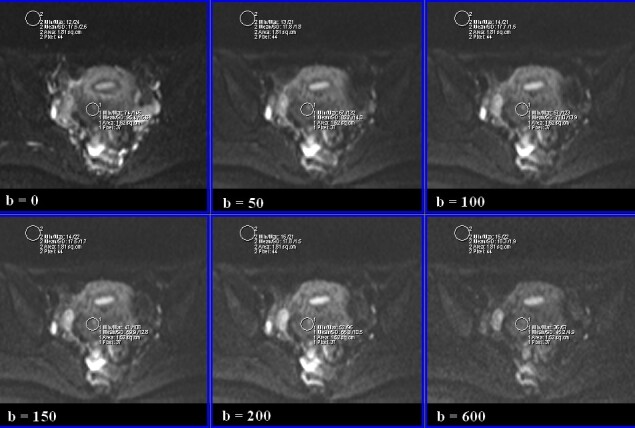

Supplement: Supplementary file 1 — Additional file 1: Figure S1. Illustrating case of signal–noise-ratio calculation at various b values DWI images. A 29-year-old female with focal adenomyoma in the posterior wall of uterine. One reviewer (H.Z.) placed the ROI1 with average area 2.54 cm2 in the center of lesion. Similarly, on the same series of pictures, ROI2 with an average area of 2.80 cm2 indicating the noise signals was also placed on the background. The SNR values in ten cases in each group were calculated. To minimum the operator bias, only one experienced operator did the whole procedure. [file 40064_2015_1635_MOESM1_ESM.jpg]

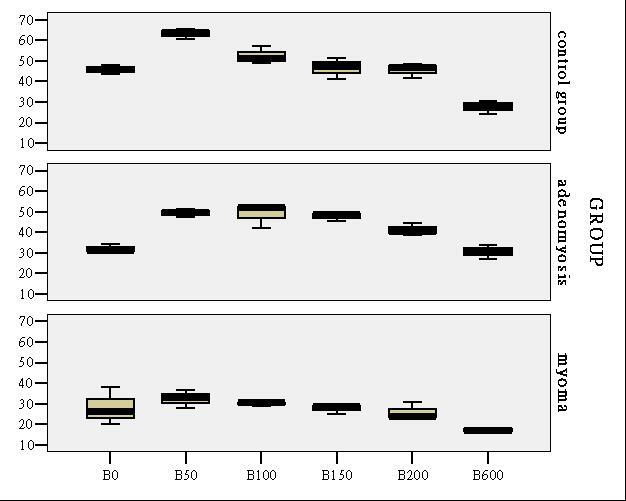

Supplement: Supplementary file 2 — Additional file 2: Figure S2. SNR values at various b values in three groups. The final SNR values for each group were 28.2 ± 9.2 at b = 0, 32.6 ± 4.3 at b = 50, 29.9 ± 0.8 at b = 100, 27.5 ± 2.1 at b = 150, 25.9 ± 4.3 at b = 200, 17.0 ± 1.0 at b = 600 for myoma and 32.1 ± 2.0 at b = 0, 49.3 ± 2.0 at b = 50, 48.9 ± 5.9 at b = 100, 47.9 ± 1.9 at b = 150, 41.4 ± 2.9 at b = 200, 30.7 ± 3.3 at b = 600 for adenomyoma and 45.8 ± 2.2 at b = 0, 63.3 ± 2.5 at b = 50, 52.5 ± 4.4 at b = 100, 46.7 ± 5.2 at b = 150,45.8 ± 3.5 at b = 200, 27.6 ± 3.3 at b = 600 for control group, respectively. [file 40064_2015_1635_MOESM2_ESM.jpg]
